# Movement Rate and Brain-Muscle Coupling in Male Footballers With and Without Hamstring Injury History

**DOI:** 10.1177/19417381251350688

**Published:** 2025-07-08

**Authors:** José Pedro Correia, Hugo Grilo, Erik Witvrouw, João R. Vaz, Sandro R. Freitas

**Affiliations:** † Laboratório de Função Neuromuscular, Faculdade de Motricidade Humana, Universidade de Lisboa, Cruz Quebrada, Portugal; ‡ CIPER, Faculdade de Motricidade Humana, Universidade de Lisboa, Cruz Quebrada, Portugal; § School of Sport, Rehabilitation and Exercise Sciences (SRES), University of Essex, Colchester, UK; ‖ Department of Rehabilitation Sciences, Ghent University Faculty of Medicine and Health Sciences, Ghent, Belgium; ¶ Egas Moniz Center for Interdisciplinary Research (CiiEM), Egas Moniz School of Health and Science, Monte da Caparica, Portugal

**Keywords:** brain-muscle coupling, fatigue, football, hamstring strain, motor control

## Abstract

**Background:**

High-speed actions constitute an important mechanism of hamstring strain injuries (HSIs) in football. These actions have a strong supraspinal base, and changes in brain activity have been noted in other musculoskeletal injuries; however, there is a lack of information about changes in brain-muscle coupling in footballers with HSI history. Therefore, this study aimed to determine whether movement speed and brain-muscle activity differ between players with and without HSI history during a high-speed knee movement task.

**Hypothesis:**

Footballers with previous HSI will show decreased knee movement rate and associated neurophysiological inhibition.

**Study Design:**

Cross-sectional study.

**Level of Evidence:**

Level 3.

**Methods:**

A total of 108 male footballers (39 with HSI history) performed a maximum-speed knee flexion-extension task over eight 10-second blocks. During this task, brain and muscle activity of knee flexors and extensors were recorded using electroencephalography (EEG) and electromyography (EMG), respectively, and the movement rate was measured.

**Results:**

Footballers with HSI history moved at a higher rate in the first half of the task. This was accompanied by higher theta and decreasing alpha EEG activity, lower rectus femoris and biceps femoris activity, and less flexor-extensor co-contraction. Conversely, there were no differences in corticomuscular coherence (CMC) between groups, but the biceps femoris showed a significantly lower CMC than all other muscles.

**Conclusion:**

The task was able to differentiate players with and without HSI history; in addition, those with previous HSI showed EEG activity patterns associated with increased task load and use of attentional resources for sensorimotor integration. EMG findings indicated players with HSI history were able to perform better despite showing overall reduced activity, especially in the rectus femoris and biceps femoris.

**Clinical Relevance:**

Neurocognitive factors may be involved in HSIs and persist even after rehabilitation, suggesting the relevance of including these factors in rehabilitation.

Despite abundant research on football-related hamstring strain injuries (HSIs), their prevalence (up to 24% of all injuries) and recurrence rate (18%) remain high.^27,28^ Previous HSI remains as the strongest risk factor.^29,76^ Sprinting is widely regarded as an HSI mechanism,^42^ with over 70% of HSIs in football occurring at high or very high horizontal speeds.^43^ Sprinting also has a great sport-specific relevance, constituting the most frequent action before goals for both the scoring and assisting player,^31^ despite accounting for only 1% to 12% of the distance covered.^26^ Accordingly, repeated sprinting is commonly used to assess hamstring performance and injury risk,^12,67^ with players with HSI history showing a greater drop in sprint speed and horizontal force production.^50,67^ Another important feature of maximal speed sprinting is the importance of intermuscular (ie, role of agonist/antagonist activation timing in step rate) and interlimb (ie, relative positioning of one limb to the other in the running cycle) coordination.^45,78^ Indeed, a bilateral difference in hamstring intermuscular coordination has been found in athletes with HSI history, even postrehabilitation.^5^ Fatigue induced by fast repetitive movements has an important supraspinal component, namely in terms of intracortical and corticospinal excitability modulation^51^; moreover, motor neuron recruitment speed and maximal motor unit discharge are the best predictors of contraction speed, and the neural drive produced in the initial phase of an explosive contraction is predominantly supraspinal.^25^

It is therefore relevant to assess isolated brain and muscle activity but also their coupling during high-speed actions in the context of HSIs. This is done using parameters such as corticomuscular coherence (CMC), which analyzes the functional brain-muscle coupling,^81^ and reflects activity synchronization at both ends of the corticospinal pathway.^71^ However, repeated sprinting is a whole-body activity with complex performance and metabolic interactions as well as multiple limiting factors, and it remains challenging to measure brain activity during sprinting. Therefore, a task that mimics the knee intermuscular and interlimb coordination and supraspinal demands of maximal sprinting but has a lower metabolic cost would be preferable to determine HSI risk and the associated brain and muscle activity. Determining neurophysiological differences in brain and muscle activity, as well as in their coupling, would be important to help ascertain whether neurocognitive rehabilitation should be included in HSI treatment.

Information about HSI-related supraspinal adaptations in athletes is scarce; nevertheless, athletes with HSI history have shown greater intracortical inhibition and lower voluntary activation.^13,14^ Furthermore, hamstring inhibition has been reported to persist postrehabilitation and to play a role in recurrence,^33,40,55^ which also highlights the relevance of looking at the impact of HSIs beyond the muscle, especially regarding central drive and motor control, in players with HSI history. The need for further research on post-HSI central nervous system adaptations has also been highlighted in the latest London International consensus on hamstring injuries.^58^ In other injuries, people with anterior cruciate ligament (ACL) tears have shown early postinjury decreased cortical excitability,^84^ greater sensorimotor cortex activity during movement,^2,36^ and decreased leg representation area volume.^32^ Lower post-ACL injury quadriceps CMC and cross-recurrence compared with uninjured controls have also been reported,^65,71^ showing that musculoskeletal injuries affect brain-muscle coupling. However, reports of such HSI-specific differences are currently lacking.

This study aimed to (1) determine whether a maximum knee movement rate task is able to differentiate football players with and without HSI history, and (2) assess differences in brain and muscle activity and brain-muscle coupling during this task between football players with and without HSI history. We hypothesized that players with HSI history would have a lower movement rate due to previous reports of HSI-associated impaired leg swing movement discrimination and altered hamstrings intermuscular coordination.^16,70^ We also hypothesized that they would show patterns of reduced brain and muscle activity (due to postinjury inhibition) and decreased CMC.

## Methods

### Study Design and Sample

This was a cross-sectional study; players were recruited from 9 participating professional and semiprofessional clubs from the Portuguese second to fourth tiers. Despite this difference in professional status, off-field activities have been shown to not influence HSI risk in nonprofessional players.^11^ Inclusion criteria were (1) playing football for ≥5 years, (2) ≥3 training sessions plus a match per week, and (3) no active injury limiting performance. Exclusion criteria were (1) playing as a goalkeeper (due to differences in HSI risk from outfield players)^35^; (2) history of knee, thigh, hip, or central nervous system structural injury or surgery; (3) practicing other sport or structured physical activity ≥2 times per week in the past 2 years; and/or (4) any condition preventing the player from completing the study protocol. This study was approved by the Institutional Review Board of Faculdade de Motricidade Humana (approval number, 15/2021); all players signed an informed consent form after all aspects of the study were explained.

### Data Collection

#### Demographics and Injury Data

Demographic and football-related data were obtained from players, including height, weight, playing position, HSI history in the previous 2 seasons, time loss due to HSIs, and time since injury. HSI was defined as any sudden onset of posterior thigh musculotendinous pain in the hamstring region that led to training or match time loss. A retrospective period of 2 seasons has been used previously in studies of football-related HSIs.^67,69^ In addition, the accuracy of hamstring injury self-reporting has been confirmed previously.^34^

#### Electroencephalography

Electroencephalography (EEG) data were collected using a 24-channel device (Vertex SC823, Meditron Eletromedicina Ltda). The Cz electrode was aligned with the midpoints of the inion and nasion in the sagittal plane and the 2 preauricular points in the coronal plane, with the remaining electrodes placed according to the International 10-20 system. The signal was online-referenced to 2 mastoid electrodes and collected at 250 Hz. A circuit impedance of ≤10 kΩ was ensured in all electrodes before data collection and a 0.1 to 70 Hz analog band-pass filter was applied by the amplifier.

#### Electromyography and Accelerometry

Surface electromyography (EMG) data were collected at 1926 Hz in the 10 Hz to 850 Hz frequency range. Electrodes were placed on the right vastus lateralis (VL), rectus femoris (RF), vastus medialis (VM), semitendinosus (ST), and biceps femoris long head (BF) of the right lower limb according to SENIAM recommendations,^38^ and secured with adhesive tape. Skin preparation included shaving and cleaning with alcohol. Accelerometry data were collected using sensors placed on the lateral malleoli at 148 Hz. We used Trigno sensors for both EMG and accelerometry data collection (Delsys).

A trigger was used to start EEG and EMG/accelerometry data collection simultaneously. The right limb was assessed in all players regardless of their dominance, as the main focus of this study was central motor control; the left hemisphere, responsible primarily for right limb movement, has been shown to specialize in controlling fast repetitive movement.^1,54^ Previous studies of lower limb movement rate have also not categorized participants according to dominance.^82,83^ Considering this functional asymmetry, the different association with dominance of the left and right hemispheres,^39^ the potentially opposing effects of injury side and dominance, and the fact that all players were symptom-free at the time of testing, we chose to place the EMG electrodes on the right side for all players. A systematic review also found evidence of no between-limb muscle activity differences in players with HSI history during maximal speed running.^60^

### Experimental Protocol

**Figure 1.**
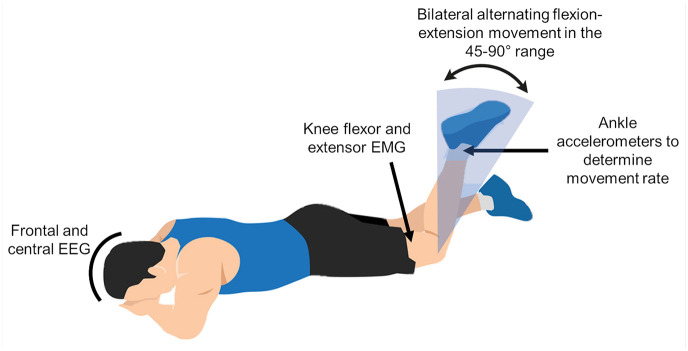
Depiction of the experimental task and data collection.

Players completed a 5-minute warm-up on a stationary bicycle at ~70 rpm without resistance. They were then placed in the prone position on a table and performed a task consisting of eight 10-second blocks of maximum-speed bilateral alternating knee flexion-extension movements between 45° and 90° interspersed by 5-second rest periods (Figure 1 and Supplemental Material 1). A familiarization period with the required range of motion was included, with soft bumpers serving as a range of motion reference during this period and removed during task performance. This method has shown nonsignificant deviations from the 45° to 90° knee range of motion^22^; the test-retest reliability of the measured parameters has also been reported.^20^ Players were instructed to always move as fast as they could, to keep their head as still as possible, and to not clench their eyes or teeth; standard encouragement was given throughout the task.

### Data Processing

Data were processed using Matlab (MathWorks Inc); data processing has been described previously in detail.^20^ Briefly, for EEG and EMG signals, the first and last seconds of each movement block were discarded to increase signal stability; for the movement rate calculation, the entire 10-second period was used. Movement rate was determined by the number of accelerometer signal peaks during each block; the presented values correspond to the movement rate of each limb.

The EEG power was calculated for the theta (4-7 Hz), alpha (8-14 Hz), beta (15-30 Hz), and gamma (31-50 Hz) bands. We calculated 2 measures of EEG power for each block: the relative power of each band as a percentage of the total power, and the band power normalized to the first block. EEG measures were calculated for the F3 and F4 (left and right frontal) and C3 and C4 (left and right central) electrodes, which correspond to areas associated with motor planning and execution and show movement rate-mediated activity.^21^ Independent component analysis (ICA) was performed using EEGLAB to remove EEG artifacts,^24^ using visual inspection to determine the components to be removed. Artifacts were identified based on their waveform and biological plausibility. If these artifacts could not be removed using ICA, artifact subspace reconstruction was used.

The EMG root mean square (RMS) was calculated as a measure of muscle activity and the values were normalized to those of the first block. Two EMG co-contraction indices (CCIs) were calculated as a measure of agonist-antagonist coordination using the percentage of overlap between the activity of the corresponding muscles^49^; one between the VL and BF (CCI_lat_) and another between the VM and ST (CCI_med_).

CMC was calculated as a measure of EEG-EMG functional coupling between the C3 electrode and the EMG signal of each muscle. The threshold for coherence significance (CT) was calculated using the following equation^82^:

$$\mathrm{CT}=1-{\left[\frac{1}{N}\left(1-\frac{\alpha }{100}\right)\right]}^{\frac{1}{\text{L}-1}}$$

where N is the number of frequency bins and is the confidence level (95% in our study). Considering the cyclic nature of our task, L was the number of cycles completed by each player in each block to adjust the CT to each participant and movement block.^82^ Two coherence outcomes were used: the total area of coherence above the CT (significant coherence) and time-frequency CMC maps as a qualitative analysis.

### Statistical Analysis

SPSS Version 25 (IBM Corp) was used for all analyses. Continuous data are presented as mean and 95% CI and categorical data are presented as frequencies and percentages. A 2-way mixed analysis of variance (ANOVA) (2 [HSI, no HSI] × 8 blocks) was performed with movement block (within-subject) and HSI history (between-subject) factors for the movement rate, EEG, EMG, and significant CMC, with post hoc Bonferroni adjustment for multiple comparisons and Greenhouse-Geisser correction if sphericity was not present. For comparing significant CMC between muscles, a 2-way repeated measures ANOVA (5 muscles × 8 blocks) was used. Effect sizes were calculated using partial eta squared (η_p_^2^) values and classified as small (0.01-0.06), medium (0.06-0.14), or large (>0.14).^18^

To assess the discriminatory ability of movement rate to differentiate players with and without HSI history, the area under the receiver operating characteristic curve (AUC) was used. The cutoff values were chosen according to the point in the curves with the greatest Youden’s index. The sensitivity, specificity, and odds ratio (OR) of HSI were calculated for the index created based on the AUC analysis, which was used to categorize players into “hares” (faster but with greater movement rate loss) and “tortoises” (slower but with lower movement rate loss). *P* values < 0.05 were considered statistically significant.

## Results

### Demographics and Injury Features

From the original population of 223 players in the 9 participating teams, a total of 129 (57.8%) participated in the study, of which 108 (48.4%) were included in the analysis (age, 24.3 ± 4.2 years; height, 1.78 ± 0.07 m; weight, 73.9 ± 7.6 kg; 15.5 ± 4.4 years of football experience; Supplemental Material 2). There were 76 right- (70.4%) and 32 left- (29.6%) lower limb dominant players. A total of 39 (36.1%) players had HSI history in the previous 2 seasons (27 on the right limb, 6 on the left limb, and 6 on both limbs), with an average time loss of 31.6 ± 25.4 days. The distribution of players and percentage of those with HSI history per playing position can be seen in Supplemental Material 3. As there were no significant differences in EMG variables (individual muscle activity and co-contraction) between left- and right-side dominant players (*P* = 0.07-0.88) or between left- and right-side injuries (*P* = 0.25-0.68) in a block × dominant/injured limb ANOVA, we proceeded with the analysis of the entire sample as a whole.

### Movement Rate

**Figure 2.**
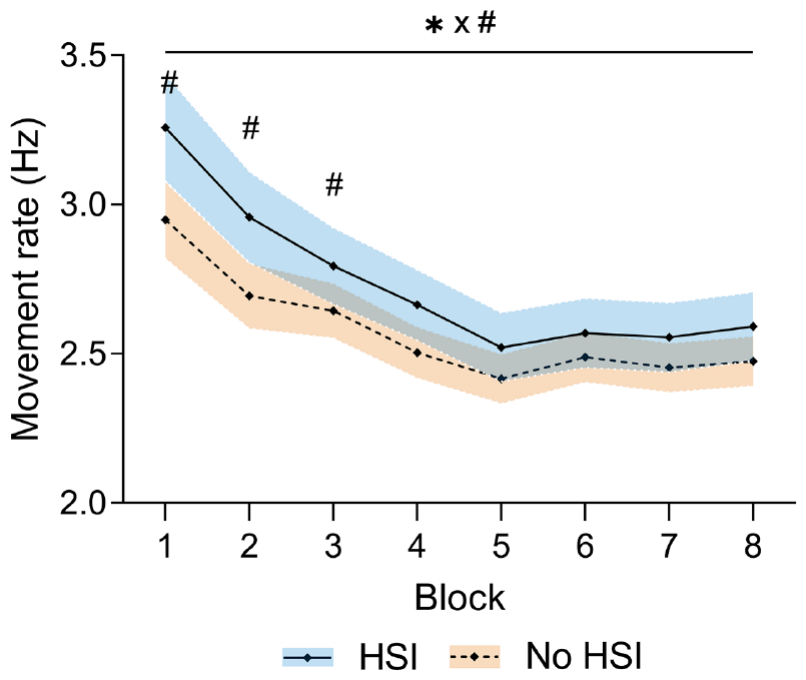
Changes in movement rate during the task. *Significant effect of the block factor; ^#^significant effect of the injury factor at individual blocks; ^×^significant block × injury interaction. Results are relative to a 2 × 8 2-way mixed ANOVA with post hoc testing. Shaded areas, 95% CI for the HSI (blue) and no HSI (orange) groups. ANOVA, analysis of variance; HSI, hamstring strain injury.

Movement rate decreased significantly during the task (*P* < 0.001) and at a different rate between groups (block × HSI interaction, *P* = 0.005) and a significant effect of the block factor (*P* < 0.001) caused by a decrease in movement rate. Players with previous HSI showed a higher movement rate (*P* = 0.03) (Figure 2, Supplemental Material 4).

**Figure 3.**
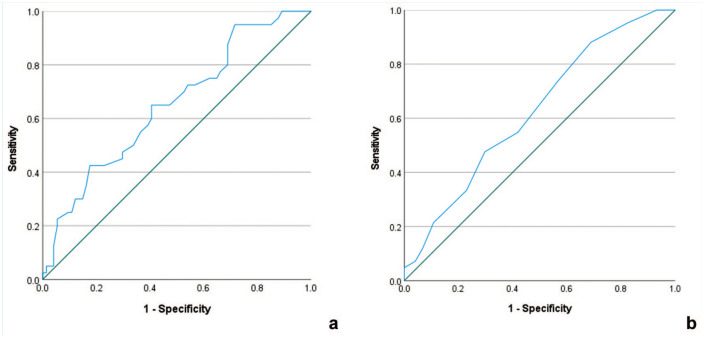
ROC curves of (a) number of cycles and (b) movement rate slope in the first 3 blocks. ROC, receiver operating characteristic.

Since the largest differences between players with and without HSI were seen in the first 3 blocks, we used the number of cycles (cutoff value, 94.5 cycles; area under the receiver operating characteristic (ROC) curve (AUC), 0.650; *P* = 0.008; Figure 3a) and the movement rate slope (cutoff value, 0.075 Hz/block; AUC, 0.626; *P* = 0.03; Figure 3b) during this period to create a movement rate index which showed a specificity of 82.6% and sensitivity of 41% for differentiating players with and without HSI in the previous 2 seasons. Players with a positive test (ie, >94.5 movement cycles and slope < –0.075 Hz per block in the first 3 blocks, the “hares”) had an OR of 3.3 (95% CI, 1.36-8.06) of having HSI history in the past 2 seasons compared with the “tortoises” (Supplemental Material 5).

### Electroencephalography

#### Relative Band Power

**Figure 4.**
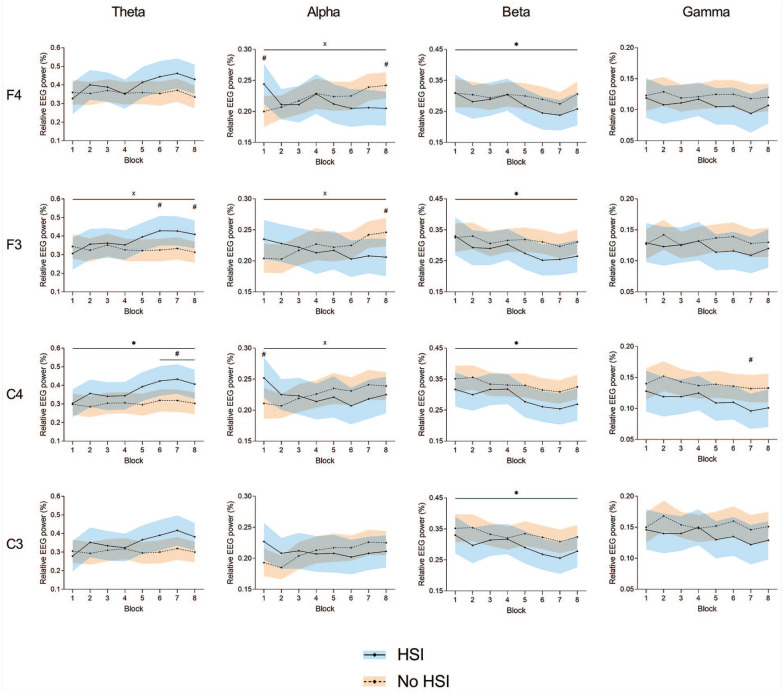
Changes in relative EEG power during the task at the frontal (F3 and F4) and central (C3 and C4) electrodes. *Significant effect of the block factor; ^#^significant between-group differences in post hoc testing; ^×^significant block × injury interaction. Results are relative to a 2 × 8 2-way mixed ANOVA with post hoc testing. Shaded areas, 95% CI for the HSI (blue) and no HSI (orange) groups. ANOVA, analysis of variance; EEG, electroencephalography; HSI, hamstring strain injury.

There were significant block × HSI history interactions at F4-alpha (*P* = 0.007), F3-theta (*P* = 0.03), F3-alpha (*P* = 0.007), and C4-alpha (*P* = 0.04). We found a significant movement block effect for F4-beta (*P* = 0.01), F3-beta (*P* = 0.02), C4-theta (*P* = 0.008), C4-beta (*P* < 0.001), and C3-beta (*P* = 0.008). No significant main effects of HSI history were found; nevertheless, post hoc testing revealed significant differences between players with and without HSI history at F4-alpha (first [*P* = 0.03] and last [*P* = 0.02] blocks), F3-theta (sixth and eighth blocks, both *P* = 0.05), F3-alpha (last block, *P* = 0.04), C4-theta (sixth to eighth block, *P* = 0.02-0.04), C4-alpha (first block, *P* = 0.05), and C4-gamma (seventh block, *P* = 0.05) (Figure 4 and Supplemental Material 6).

#### Normalized Band Power

**Figure 5.**
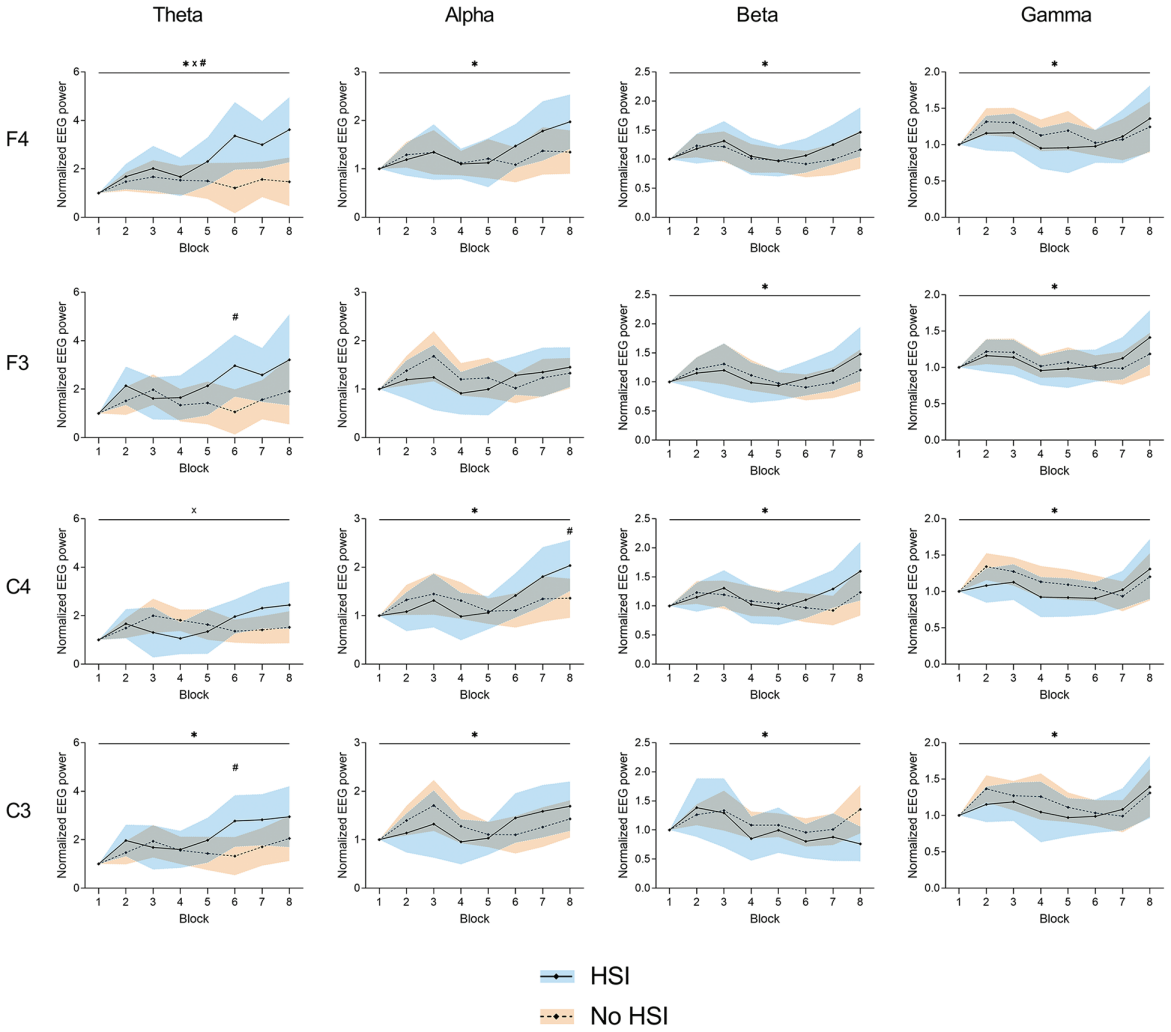
Changes in normalized EEG power during the task at the frontal (F3 and F4) and central (C3 and C4) electrodes. *Significant effect of the block factor; ^#^significant effect of the injury factor (above top line) or between-group differences in post hoc testing (above corresponding block); ^×^significant block × injury interaction. Results are relative to a 2 × 8 2-way mixed ANOVA with post hoc testing. Shaded areas, 95% CI for the HSI (blue) and no HSI (orange) groups. ANOVA, analysis of variance; EEG, electroencephalography; HSI, hamstring strain injury.

Significant movement block × HSI history interactions were found at F4-theta (*P* = 0.01) and C4-theta (*P* = 0.04). A significant movement block effect was seen at all electrodes: F4 (all frequency bands, *P* = 0.004-0.05), F3 (beta and gamma, *P* = 0.005-0.02), C4 (alpha, beta, and gamma, *P* = 0.004-0.01), and C3 (all frequency bands, *P* = 0.004-0.02). There was a significant effect of HSI history at F4-theta (*P* = 0.02). Post hoc testing also revealed differences between players with and without HSI history at F3-theta (sixth block, *P* = 0.02), C4-alpha (eighth block, *P* = 0.05), and C3-theta (sixth block, *P* = 0.03) (Figure 5 and Supplemental Material 7).

### Corticomuscular Coherence

**Figure 6.**
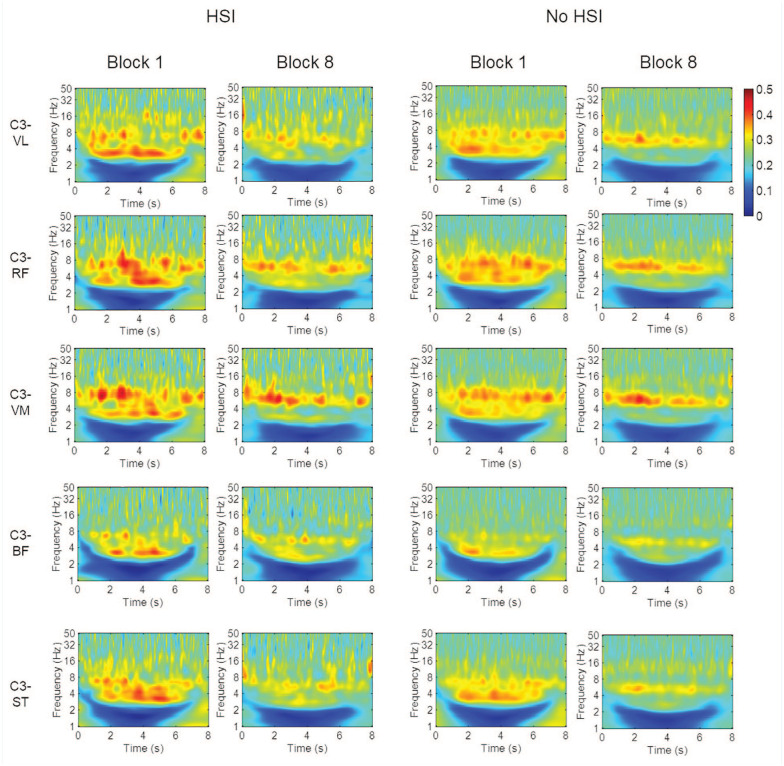
Time-frequency CMC maps for the first and last blocks. BF, biceps femoris; CMC corticomuscular coherence; RF, rectus femoris; ST, semitendinosus; VL, vastus lateralis; VM, vastus medialis.

The time-frequency maps show a loss in coherence from the first to the last block in all muscles and in both groups. The knee extensors showed stronger CMC than the flexors, and the 2 frequency zones with the strongest coherence were around 3 to 4 and 8 to 10 Hz. There was a greater loss of coherence in the 3 to 4 Hz range from the first to the last block (Figure 6).

**Figure 7.**
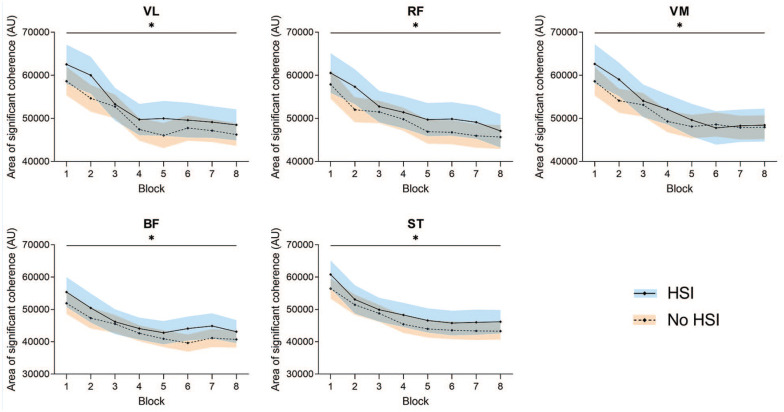
Changes in the area of significant CMC during the task. *Significant effect of the block factor. Results are relative to a 2 × 8 2-way mixed ANOVA with post hoc testing. Shaded areas, 95% CI for the HSI (blue) and no HSI (orange) groups. ANOVA, analysis of variance; AU, arbitrary units; BF, biceps femoris; CMC corticomuscular coherence; HSI, hamstring strain injury; RF, rectus femoris; ST, semitendinosus; VL, vastus lateralis; VM, vastus medialis.

In the significant CMC area analysis (Figure 7, Supplemental Material 8), there were no significant block × HSI interactions (*P* = 0.25-0.90) or any pairs with a significant HSI history effect (*P* = 0.15-0.33). Conversely, all EEG-EMG pairs showed a significant effect of the block factor caused by a decrease in coherence (all *P* < 0.001). The C3-BF pair showed a significantly lower CMC area than any other muscle (*P* < 0.001; Supplemental material 9).

### Electromyography

Significant decreases were seen in the normalized RMS of all tested muscles during the task (*P* = 0.009 to *P* < 0.001). Significant differences between players with and without HSI history were seen for the RF (*P* = 0.04) and BF (*P* = 0.04), with previously injured players showing lower normalized muscle activity.

**Figure 8.**
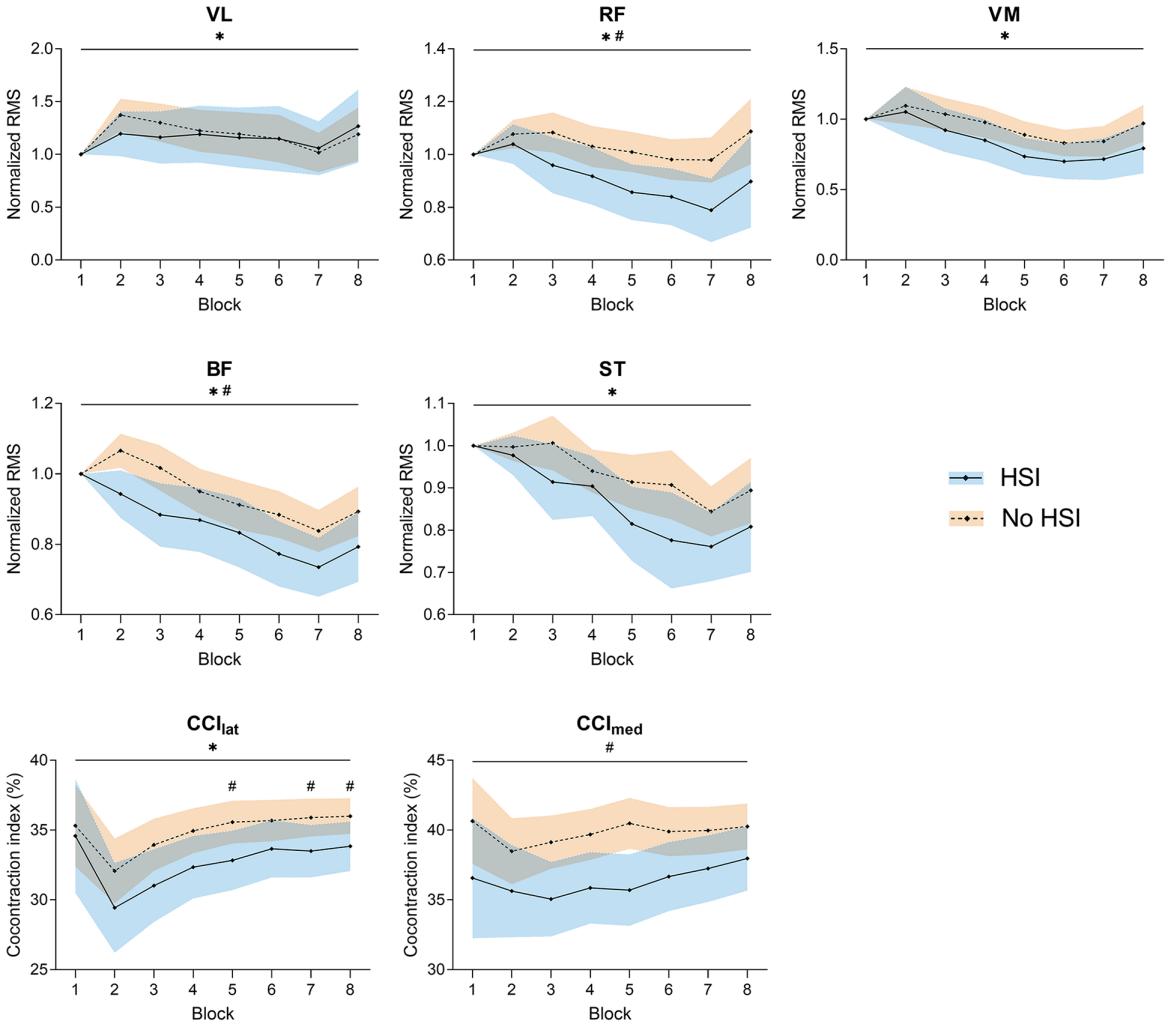
Changes in normalized EMG activity and co-contraction indices during the task. *Significant effect of the block factor; ^#^significant effect of the injury factor (above top line) or between-group differences in post hoc testing (above corresponding block); ^×^significant block × injury interaction. Results are relative to a 2 × 8 2-way mixed ANOVA with post hoc testing. Shaded areas, 95% CI for the HSI (blue) and no HSI (orange) groups. BF, biceps femoris; CCI_lat_, lateral co-contraction index; CCI_med_, medial co-contraction index; EMG, electromyography; RF, rectus femoris; RMS, root mean square; ST, semitendinosus; VL, vastus lateralis; VM, vastus medialis.

As for the co-contraction indices, there was a main effect of the block factor for CCI_lat_ (*P* < 0.001), caused by an increase from the second block onwards, but not for CCI_med_. Conversely, there was a significant effect of HSI history for CCI_med_ (*P* = 0.02), with lower values for previously injured players, but not for CCI_lat_. Nevertheless, post hoc testing revealed that players with HSI history had a significantly lower CCI_lat_ in the fifth, seventh, and eighth blocks (Figure 8, Supplemental Material 10).

## Discussion

We found significant differences in movement rate between players with and without HSI history, with previously injured players showing a greater rate, especially in the first half of the task (Figure 2). Alongside this, players with HSI history showed increased EEG theta activity in the second half of the task, decreasing alpha activity, lower RF and BF EMG activity, and a lesser degree of co-contraction (Figures 4-6 and 8).

The findings contradict our original hypothesis of lower movement rate and greater co-contraction in players with HSI history, although this association between greater movement speed and HSI history is not unprecedented. Previously injured players have shown a higher maximal theoretical sprinting velocity,^43^ and footballers with a “fast” muscle fiber typology have shown a significantly greater prospective HSI risk.^48^ In addition, ATPase and FXYD1 expression (as a proxy of muscle composition) have also been associated positively with high-speed running/sprinting distance in football.^52^ These findings help understand our results both in terms of movement rate and EMG activity. The better performance of players with HSI history may be caused by a greater percentage of fast-twitch fibers, which would lead to a greater drop in EMG amplitude, since these fibers are more fatigable and have been shown to be recruited preferentially during high-speed contractions.^78^ Similarly to the “jumper’s knee paradox,” where athletes able to jump higher are at an increased risk for patellar tendinopathy,^77^ a “hare paradox” may exist for HSIs. In this case, players who show greater ability to develop higher movement rates/sprint speed are shifted to roles where this attribute is more relevant, increasing their sprint and high-speed actions exposure and HSI risk. Accordingly, in our study, lateral defenders (55%), forwards (45.4%), and wingers (35.3%) (Supplemental Material 3), who all perform a significantly greater sprinting distance in elite football matches,^26^ were among the positions with greater HSI prevalence.

Performance differences between players with and without HSI history were more evident in the first 3 blocks, with both groups stabilizing movement rates by the fifth block, leading to the significant block × HSI history interaction (Figure 2). Our index, combining the number of cycles and the loss in movement rate during the first 3 blocks, showed high specificity but low sensitivity in differentiating players with and without HSI history, making it more useful for identifying low-risk players (“tortoises”). The usefulness of this field-applicable index should be confirmed prospectively. Nevertheless, players with a positive index (“hares”) were over 3 times more likely to have HSI history. This index was derived from accelerometry values, which are obtained more easily in the field than EMG or EEG parameters, highlighting its applicability.

Players with HSI history showed increased frontal EEG theta activity, associated with sensorimotor integration and attention allocation,^7,23^ during the second half of the task (Figure 5). Fatigue decreases the knee joint position sense ability,^61^ which may be driven by changes in muscle spindle activity.^64^ This increase in frontal theta activity in players with HSI history could be driven by additional attentional requirements due to muscle spindle activity impairment by the combined effects of previous injury and fatigue (since this was seen in the second half of the task), affecting afferent input. Although speculative, this hypothesis is supported by previous findings of region-specific proprioceptive and spatial processing impairments in athletes with previous HSI and increased theta activity in people with reconstructed ACL during force-matching and angle reproduction tasks,^8,9,73^ which were attributed to a greater neurocognitive involvement. Moreover, it has been reported that ACL injuries lead to knee proprioception deficits and brain activity differences.^15,36^ Our similar findings of increased frontal theta activity therefore suggest that muscle injuries may also be associated with sensorimotor processing differences as seen post-ACL tears.

As for the alpha band, players with previous HSI showed a continuous decrease in relative frontal and central alpha activity, while noninjured players had a significant continuous decrease, leading to the significant HSI × movement block interactions (Figure 4). Alpha power has been associated with motor planning and execution and found to decrease with fatigue.^6,7^ However, movement rate did not show an association with these opposing patterns of alpha activity. It is therefore unlikely that differences in fatigue or performance levels led to these findings. Since alpha activity suppression has been associated with increased task load/difficulty,^47,62^ the combined findings of higher theta and lower alpha activity in HSI players suggest additional attentional resource use for sensorimotor integration (increasing theta activity) and a greater cognitive load in these players (decreasing alpha activity). This opposing but associated behavior of theta and alpha activity as a sign of increased workload has been reported previously,^17,62^ but we cannot conclude whether it represents a cause or a consequence of HSIs. Regardless of the direction of this association, these findings imply that players with previous HSI display a higher cognitive load during a maximal movement task, thus decreasing their cognitive reserve. A higher cognitive load has been shown to influence actions such as kicking,^30^ and to increase noncontact lower limb injury risk due to altered biomechanical movement patterns.^10,63^ We therefore expand this knowledge about increased risk to the context of HSIs, specifically during an action that constitutes a frequent injury mechanism.

The C3-BF pair showed significantly lower CMC than any other muscle (Supplemental Material 9). This is interesting considering that the BF is more commonly affected by HSIs in football.^4,27^ However, this lower coherence was seen both in players with and without HSI history. This may be worth exploring further. Contrary to what has been noted post-ACL injury,^65,71^ athletes with previous HSI did not show lower CMC. This may have been due either to HSIs causing less disruption in brain-muscle communication compared with ACL injuries or to task differences, as dynamic tasks (such as in our study) have been shown to abolish 15 to 30 Hz CMC.^46^ Fast movement tasks have also shown greater CMC at <12 Hz,^19^ which was confirmed by our findings of stronger coherence at 3 to 10 Hz in this and a previous study.^20^ Since CMC is reported to decrease with fatigue,^81^ it could also be that fatigue had a greater influence than HSI history in the changes in CMC, thus preventing the detection of between-group differences.

The increase in theta activity was accompanied by decreased BF and RF activity compared with players without HSI history (Figure 8), despite the fact that the latter showed worse task performance. Lower EMG activity coupled with higher theta and lower alpha EEG activity, as seen in the HSI group, has also been associated with a predominant external attentional focus, which indicates a greater cortical inhibitory control and sensorimotor processing ability.^57,72^ Attentional control strategies and sensorimotor processing requirements may therefore contribute to the electrophysiological findings associated with improved task performance in the HSI group. EMG studies in athletes with previous HSI have also shown lower BF activity,^3,40,56^ which is commonly attributed to persistent postrehabilitation inhibition.^13,33^ We add that a hamstring antagonist, the RF, also showed significantly lower activity, highlighting the relevance of agonist-antagonist coupling in fast cyclic tasks such as the one used here. Indeed, a significant decrease in the activity of biarticular antagonists (such as the RF in our case) has been noted in high-intensity cyclical efforts as an adjustment to fatigue of the power producers (the knee flexors in our task) ^37^; RF/BF coordination during sprinting has also been shown to affect maximum step frequency.^45^ The degree of agonist-antagonist co-contraction plays a significant role in the maximum movement rate,^66^ and RF/BF co-contraction contributes to maintaining posture during the early ground contact phase.^59^ It therefore makes sense that players with HSI history showed decreased knee co-contraction in association with their higher movement rate. Conversely, increased knee stiffness (due to greater quadriceps-hamstrings co-contraction) has a protective effect against potentially hazardous loads,^74^ which may explain the greater co-contraction of players without HSI history.

Our study showed the usefulness of a simple high-speed movement task to differentiate players with and without previous HSI and adds to existing evidence regarding the relevance of high-speed actions in HSI risk. We show that differences in brain and muscle activity are present in players with previous HSI in association with fatigue induced by high-speed movement; notably, these differences were present regardless of the injured limb or the time since injury. If both the discriminating nature of the test and the brain and muscle activity differences are confirmed prospectively, this task may be used for evaluation and training throughout the season to detect and address players at risk, as well as to monitor rehabilitation/readiness, which would have a significant application in screening and recovery protocols. Our EEG findings also alert to the importance of monitoring attentional/neurocognitive resources in players with previous HSI as part of rehabilitation, prevention, and recurrence management programs, as suggested for other injuries,^41,63^ especially as they were present even after returning to play. This suggests the need for interventions aimed at promoting effective cortical resource usage and improved attentional control both during and after rehabilitation. These include cognitive-motor dual-task paradigms and neurofeedback monitoring,^53,68,75,80^ which have shown beneficial long-term effects on athletes’ attentional control and resource efficiency.

## Limitations

This study presents considerable limitations. First, the retrospective design means we cannot infer causality; injuries were also self-reported, which may be influenced by recall bias. Second, our task does not replicate footballing demands; thus, the outcomes do not represent ecological player behavior. Third, the results should not be generalized to female players and to different competitive levels. Fourth, the task characteristics mean that some signal noise and artifacts can be expected despite the best signal processing efforts. Fifth, we did not consider the influence of leg length/weight differences between players; however, since there were no differences in height or weight, this is unlikely to have affected the results. Sixth, we were not able to determine the injured muscle in all cases, which prevented a more specific analysis. Finally, since we collected EMG data from only one limb, it is unclear what interlimb differences may have been present.

## Conclusion

The simple movement rate task used in this study was able to differentiate football players with and without HSI history and therefore has the potential for field application. Surprisingly, the “hares” showed a higher HSI history prevalence. In association with the movement rate differences, footballers with HSI history showed higher theta EEG power in the second half of the task, an opposing alpha EEG power pattern, lower EMG flexor and RF activity, and a lower degree of co-contraction. However, no differences in brain-muscle coupling were found between groups. Our findings highlight the presence of central and peripheral motor control differences in footballers with HSI history compared with those without.

## Supplemental Material

sj-docx-1-sph-10.1177_19417381251350688 – Supplemental material for Movement Rate and Brain-Muscle Coupling in Male Footballers With and Without Hamstring Injury History Supplemental material, sj-docx-1-sph-10.1177_19417381251350688 for Movement Rate and Brain-Muscle Coupling in Male Footballers With and Without Hamstring Injury History by José Pedro Correia, Hugo Grilo, Erik Witvrouw, João R. Vaz and Sandro R. Freitas, in Sports Health
