# Supplementary material for: Movement Rate and Brain-Muscle Coupling in Male Footballers With and Without Hamstring Injury History
Source: Sports Health. 2025 Jul 8;18(2):378–90. doi: 10.1177/19417381251350688 (PMC12237958; doi:10.1177/19417381251350688)
Supplement: sj-docx-1-sph-10.1177_19417381251350688 – Supplemental material for Movement Rate and Brain-Muscle Coupling in Male Footballers With and Without Hamstring Injury History [file sj-docx-1-sph-10.1177_19417381251350688.pdf]

Supplemental material 2. Study flowchart

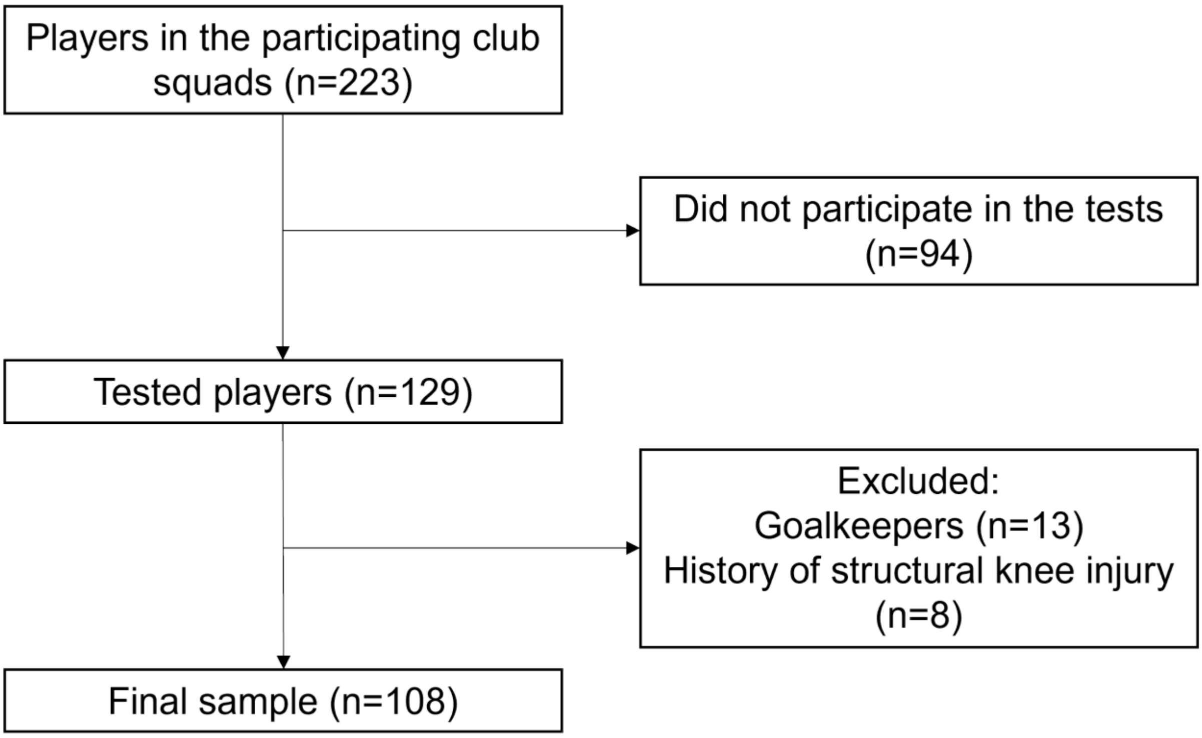

Supplemental material 3. Distribution of players per playing position

**Position**	**n (%)**	**HSI history**		
		**n**	**% of all HSIs**	**Prevalence within the position**
Lateral defenders	20 (18.5%)	11	28.2%	55%
Central defenders	26 (24.1%)	8	20.5%	30.7%
Defensive midfielders	8 (7.4%)	2	5.1%	25%
Central midfielders	15 (13.9%)	3	7.7%	20%
Attacking midfielders	11 (10.2%)	4	10.3%	36.3%
Wingers	17 (15.7%)	6	15.4%	35.3%
Forwards	11 (10.1%)	5	12.8%	45.4%
**Total**	108	39		

Supplemental material 4. Movement rate findings

Variable		Block 1	2	3	4	5	6	7	8	Within subjects p	ηp²	Power	Between subjects p	ηp²	Power	Interaction p	ηp²	Power
**MR**	HSI	3.26	2.96	2.79	2.66	2.52	2.57	2.56	2.59	<0.001	0.568	>0.99	0.031	0.044	0.58	0.005	0.046	0.85
	No HSI	2.95	2.69	2.64	2.50	2.42	2.49	2.45	2.48									
**RPE**	HSI	4.39	5.88	7.15	7.88	8.52	9.09	9.42	9.67	<0.001	0.792	>0.99	0.18	0.019	0.27	0.50	0.007	0.17
	No HSI	4.29	5.40	6.48	7.46	8.05	8.63	8.95	9.31									

MR, movement rate; HSI, hamstring strain injury; ηp², partial eta squared

Supplemental material 5. Movement rate index and HSI history crosstable

		Movement rate index	
		Positive	Negative
HSI history	Yes	16	23
	No	12	57

Supplemental material 6. Relative EEG power findings

Electrode	Band		Block 1	2	3	4	5	6	7	8	Within subjects p	ηp²	Power	Between subjects p	ηp²	Power	Interaction p	ηp²	Power
**F4**	Theta	HSI	0.33	0.40	0.39	0.35	0.41	0.44	0.46	0.43	0.073	0.026	0.68	0.285	0.015	0.186	0.060	0.027	0.70
		No HSI	0.36	0.35	0.37	0.36	0.36	0.35	0.37	0.34									
	Alpha	HSI	0.24	0.21	0.21	0.23	0.21	0.21	0.21	0.21	0.648	0.009	0.28	0.56	0.004	0.088	0.007	0.036	0.90
		No HSI	0.2	0.21	0.22	0.23	0.22	0.23	0.24	0.24									
	Beta	HSI	0.31	0.28	0.29	0.30	0.27	0.25	0.24	0.26	0.013	0.035	0.85	0.40	0.009	0.13	0.49	0.011	0.31
		No HSI	0.31	0.30	0.29	0.31	0.30	0.29	0.27	0.31									
	Gamma	HSI	0.12	0.11	0.11	0.12	0.11	0.11	0.09	0.11	0.63	0.008	0.23	0.36	0.010	0.15	0.76	0.006	0.18
		No HSI	0.12	0.13	0.12	0.12	0.13	0.13	0.12	0.12									

**F3**	Theta	HSI	0.31	0.36	0.36	0.35	0.40	0.43	0.43	0.41	0.22	0.017	0.50	0.23	0.018	0.23	0.031	0.030	0.78
		No HSI	0.34	0.32	0.35	0.33	0.32	0.33	0.33	0.31									
	Alpha	HSI	0.24	0.23	0.22	0.21	0.22	0.20	0.21	0.21	0.93	0.004	0.13	0.63	0.003	0.08	0.007	0.037	0.90
		No HSI	0.20	0.20	0.22	0.23	0.22	0.23	0.24	0.25									
	Beta	HSI	0.33	0.29	0.29	0.30	0.27	0.25	0.26	0.27	0.017	0.034	0.83	0.26	0.016	0.20	0.35	0.014	0.39
		No HSI	0.33	0.33	0.31	0.32	0.32	0.31	0.30	0.31									
	Gamma	HSI	0.13	0.12	0.13	0.13	0.11	0.12	0.11	0.12	0.66	0.008	0.21	0.49	0.006	0.11	0.43	0.12	0.32
		No HSI	0.13	0.14	0.13	0.13	0.14	0.14	0.13	0.13									
**C4**	Theta	HSI	0.30	0.36	0.34	0.34	0.39	0.42	0.43	0.41	0.008	0.038	0.89	0.077	0.039	0.42	0.10	0.022	0.74
		No HSI	0.30	0.29	0.30	0.31	0.30	0.32	0.32	0.30									
	Alpha	HSI	0.25	0.23	0.22	0.21	0.22	0.21	0.22	0.23	0.62	0.009	0.28	0.82	0.001	0.056	0.039	0.028	0.77
		No HSI	0.21	0.21	0.22	0.23	0.24	0.23	0.24	0.24									
	Beta	HSI	0.32	0.30	0.32	0.32	0.28	0.26	0.25	0.27	0.001	0.049	0.95	0.11	0.031	0.35	0.53	0.010	0.36
		No HSI	0.35	0.36	0.33	0.33	0.33	0.32	0.31	0.33									
	Gamma	HSI	0.13	0.12	0.12	0.13	0.11	0.11	0.10	0.10	0.065	0.026	0.69	0.11	0.033	0.37	0.58	0.009	0.27
		No HSI	0.14	0.15	0.14	0.14	0.14	0.14	0.13	0.13									
**C3**	Theta	HSI	0.28	0.35	0.33	0.32	0.37	0.39	0.42	0.38	0.082	0.024	0.65	0.22	0.019	0.24	0.085	0.024	0.68
		No HSI	0.31	0.29	0.31	0.32	0.30	0.30	0.32	0.30									
	Alpha	HSI	0.23	0.21	0.21	0.21	0.21	0.20	0.21	0.21	0.49	0.011	0.34	0.98	<0.001	0.050	0.083	0.024	0.68
		No HSI	0.19	0.19	0.20	0.21	0.22	0.22	0.23	0.23									
	Beta	HSI	0.33	0.30	0.31	0.32	0.29	0.27	0.26	0.28	0.008	0.040	0.88	0.17	0.024	0.28	0.44	0.012	0.32
		No HSI	0.35	0.35	0.33	0.32	0.34	0.32	0.31	0.32									
	Gamma	HSI	0.15	0.14	0.14	0.15	0.13	0.14	0.12	0.13	0.33	0.014	0.40	0.29	0.014	0.18	0.51	0.011	0.30
		No HSI	0.15	0.17	0.15	0.15	0.15	0.16	0.15	0.15									

HSI, hamstring strain injury; ηp^2, partial eta squared

Supplemental material 7. Normalized EEG power findings

			**Block**								**Within subjects**			**Between subjects**			**Interaction**		
**Electrode**	Band		1	2	3	4	5	6	7	8	p	ηp^2	Power	p	ηp^2	Power	p	ηp^2	Power
**F4**	Theta	HSI	1	1.68	2.02	1.67	2.31	3.36	3.00	3.62	0.004	0.056	0.90	0.021	0.08	0.64	0.013	0.047	0.83
		No HSI	1	1.47	1.67	1.53	1.50	1.21	1.57	1.47									

	Alpha	HSI	1	1.19	1.35	1.10	1.13	1.47	1.78	1.97	0.004	0.046	0.91	0.44	0.008	0.12	0.24	0.018	0.47
		No HSI	1	1.29	1.34	1.12	1.21	1.09	1.37	1.35									
	Beta	HSI	1	1.18	1.31	1.05	0.97	1.06	1.25	1.46	0.010	0.040	0.86	0.47	0.007	0.11	0.58	0.009	0.25
		No HSI	1	1.23	1.22	1.01	0.98	0.92	0.99	1.16									
	Gamma	HSI	1	1.16	1.16	0.95	0.96	0.98	1.11	1.36	0.049	0.030	0.69	0.56	0.004	0.090	0.63	0.008	0.21
		No HSI	1	1.32	1.30	1.13	1.19	1.03	1.07	1.24									
**F3**	Theta	HSI	1	2.14	1.62	1.65	2.14	2.97	2.59	3.21	0.064	0.032	0.63	0.11	0.036	0.36	0.21	0.021	0.43
		No HSI	1	1.52	1.98	1.34	1.43	1.06	1.57	1.91									
	Alpha	HSI	1	1.20	1.24	0.92	1.00	1.29	1.35	1.45	0.14	0.022	0.56	0.64	0.003	0.075	0.45	0.012	0.31
		No HSI	1	1.38	1.68	1.20	1.23	1.02	1.24	1.33									
	Beta	HSI	1	1.15	1.20	0.99	0.94	1.06	1.20	1.48	0.020	0.035	0.80	0.80	0.001	0.060	0.48	0.011	0.29
		No HSI	1	1.22	1.31	1.12	0.98	0.91	0.99	1.21									
	Gamma	HSI	1	1.16	1.14	0.96	0.98	1.02	1.13	1.41	0.005	0.046	0.89	0.90	<0.001	0.052	0.46	0.012	0.29
		No HSI	1	1.22	1.21	1.02	1.07	1.00	0.99	1.19									
**C4**	Theta	HSI	1	1.67	1.31	1.06	1.35	1.96	2.31	2.44	0.093	0.031	0.63	0.67	0.003	0.071	0.042	0.038	0.74
		No HSI	1	1.49	2.00	1.81	1.63	1.36	1.42	1.52									
	Alpha	HSI	1	1.08	1.31	0.98	1.06	1.42	1.81	2.03	0.004	0.052	0.90	0.63	0.003	0.076	0.079	0.028	0.62
		No HSI	1	1.33	1.45	1.31	1.09	1.11	1.35	1.36									
	Beta	HSI	1	1.15	1.31	1.03	0.95	1.11	1.29	1.60	0.009	0.046	0.84	0.514	0.006	0.099	0.21	0.019	0.43
		No HSI	1	1.23	1.19	1.08	1.04	0.97	0.92	1.24									
	Gamma	HSI	1	1.08	1.13	0.92	0.92	0.90	1.03	1.31	0.012	0.043	0.82	0.43	0.008	0.12	0.33	0.015	0.35
		No HSI	1	1.34	1.28	1.13	1.09	1.04	0.93	1.20									
**C3**	Theta	HSI	1	1.97	1.68	1.60	1.98	2.77	2.82	2.95	0.004	0.057	0.91	0.13	0.036	0.33	0.17	0.025	0.52
		No HSI	1	1.47	1.94	1.56	1.43	1.32	1.71	2.05									
	Alpha	HSI	1	1.14	1.32	0.96	1.03	1.45	1.59	1.69	0.022	0.036	0.79	0.95	<0.001	0.050	0.22	0.019	0.46
		No HSI	1	1.40	1.71	1.28	1.11	1.10	1.26	1.43									
	Beta	HSI	1	1.10	1.25	0.99	0.93	1.06	1.19	1.49	0.009	0.043	0.85	0.94	<0.001	0.051	0.60	0.008	0.21
		No HSI	1	1.26	1.33	1.09	1.08	0.96	1.01	1.36									
	Gamma	HSI	1	1.15	1.19	1.05	0.97	0.99	1.08	1.39	0.011	0.041	0.84	0.62	0.003	0.079	0.59	0.009	0.22
		No HSI	1	1.37	1.27	1.26	1.11	1.03	0.99	1.31									

HSI, hamstring strain injury; ηp^2, partial eta squared

Supplemental material 8. Significant corticomuscular coherence findings

		Block								Within subjects			Between subjects			Interaction		
**Pair**		1	2	3	4	5	6	7	8	p	ηp^2	Power	p	ηp^2	Power	p	ηp^2	Power
**C3-VL**	HSI	62545	60012	53277	49726	49991	49592	49149	48495	<0.001	0.28	>0.99	0.15	0.22	0.30	0.53	0.009	0.31
	No HSI	58648	54661	52744	47438	46026	47764	47155	46234									
**C3-RF**	HSI	60531	57339	52751	51424	49706	49866	49109	47092	<0.001	0.23	>0.99	0.17	0.021	0.28	0.64	0.007	0.22
	No HSI	57890	52008	51494	49812	46907	46757	45953	45665									
**C3-VM**	HSI	62634	59047	54069	52082	49601	47784	48288	48438	<0.001	0.25	>0.99	0.33	0.011	0.16	0.25	0.014	0.48
	No HSI	58615	54109	53141	49287	48107	48576	47861	47955									
**C3-BF**	HSI	55386	50437	46194	44074	42799	44091	44883	43126	<0.001	0.23	>0.99	0.17	0.021	0.28	0.70	0.007	0.23
	No HSI	51916	47329	45508	42616	40932	39613	41148	40718									
**C3-ST**	HSI	60811	53077	49908	48290	46585	45775	46024	46198	<0.001	0.28	>0.99	0.18	0.019	0.27	0.90	0.004	0.14
	No HSI	56455	51452	48772	45408	43984	43544	43343	43277									

VL, vastus lateralis; RF, rectus femoris; VM, vastus medialis; BF, biceps femoris; ST, semitendinosus; HSI, hamstring strain injury; ηp^2, partial eta squared

Supplemental material 9. Changes in the area of significant corticomuscular coherence in the whole sample between the C3 electrode and each muscle (A) and of the biceps femoris in comparison to the average of other muscles (B).

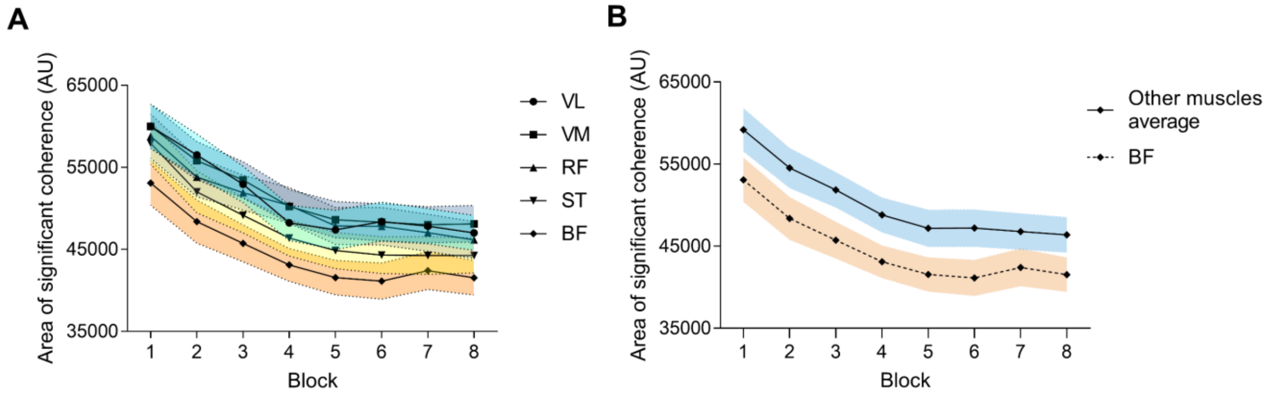

Supplemental material 10. Normalized EMG and co-contraction findings

		Block								Within subjects			Between subjects			Interaction		
**Variable**		1	2	3	4	5	6	7	8	p	ηp^2	Power	p	ηp^2	Power	p	ηp^2	Power
**VL**	HSI	1	1.20	1.16	1.19	1.16	1.15	1.06	1.27	0.013	0.036	0.83	0.79	0.001	0.058	0.64	0.007	0.20
	No HSI	1	1.37	1.30	1.22	1.19	1.15	1.02	1.19									
**RF**	HSI	1	1.04	0.96	0.92	0.86	0.84	0.79	0.90	<0.001	0.067	0.97	0.029	0.050	0.60	0.088	0.023	0.55
	No HSI	1	1.08	1.08	1.03	1.01	0.98	0.98	1.09									
**VM**	HSI	1	1.05	0.92	0.85	0.74	0.70	0.72	0.79	<0.001	0.135	>0.99	0.13	0.026	0.33	0.43	0.011	0.29
	No HSI	1	1.10	1.04	0.98	0.89	0.83	0.84	0.97									
**BF**	HSI	1	0.94	0.88	0.87	0.83	0.77	0.74	0.79	<0.001	0.183	>0.99	0.023	0.056	0.63	0.26	0.015	0.41
	No HSI	1	1.07	1.02	0.95	0.91	0.88	0.84	0.89									
**ST**	HSI	1	0.98	0.91	0.90	0.82	0.78	0.76	0.81	<0.001	0.134	>0.99	0.058	0.039	0.48	0.28	0.014	0.41
	No HSI	1	1.00	1.01	0.94	0.91	0.91	0.84	0.89									
**CCI_{lat}**	HSI	34.58	29.44	31.02	32.35	32.83	33.65	33.49	33.84	<0.001	0.098	0.98	0.091	0.034	0.39	0.62	0.006	0.13
	No HSI	35.32	32.07	33.94	34.95	35.57	35.68	35.90	36.00									
**CCI_{med}**	HSI	36.56	35.63	35.05	35.86	35.69	36.67	37.24	37.97	0.16	0.021	0.41	0.020	0.060	0.65	0.57	0.007	0.16
	No HSI	40.65	38.49	39.14	39.68	40.48	39.89	39.96	40.26									

VL, vastus lateralis; RF, rectus femoris; VM, vastus medialis; BF, biceps femoris; ST, semitendinosus; CCI, co-contraction index; HSI, hamstring strain injury; ηp^2, partial eta squared
